# The Craving and Excitement of Social Networking Sites Addicts: Based on Cue-Reactivity

**DOI:** 10.3389/fpsyg.2019.01717

**Published:** 2019-08-09

**Authors:** Yexi Leng, Xi He, Baijie Zhu, Ping Li, Chuan Xiao, Weiqi He

**Affiliations:** ^1^Research Center of Brain and Cognitive Neuroscience, Liaoning Normal University, Dalian, China; ^2^Tanghu School, Yibin, China

**Keywords:** social networking, addiction, cue-reactivity, craving, excitability

## Abstract

Everyone benefits from social networking as a daily tool, but there are potential addictions. However, little is known about the craving and excitability of social networking sites addiction, and mode of change in psychological craving. The study consisted of two experiments that used a cue-reactivity paradigm to study the cravings and excitement of social networking sites (SNSs) addiction and the changing regulars in cravings. Sixty subjects were divided into a high-score group and a low-score group. In Experiment 1, all subjects evaluated word clues. The results showed that the SNS-related clues only induced the craving and excitability of the high-score group, but not the low-score group, and the craving fluctuated. Furthermore, in Experiment 2, image clues were used. The results showed that the craving induced by an image clue is significantly higher than the craving induced by a word clue, and there is no difference in excitability. Taken together, our findings suggest the SNS-related stimulation, especially image clues, could significantly induce subjects for the craving and excitability of social networks, and the craving fluctuates.

## Introduction

The development of communication technology and the rapidly increasing use of smartphones and computers have made the Internet an indispensable part of modern society. Social networking sites represent an important form of network activity with an exponential rise in usage since 1997. SNSs are primarily used for both making new friends and maintaining and enhancing existing relationships between friends (Boyd and Ellison, 2007; Lenhart and Madden, 2007).

Recently, Internet Addiction Disorder (IAD) has received increased attention, and SNS addiction is considered one of the five types of IAD (Young, 1999, 2017). IAD is similar to chemical and behavioral addictions in that it also conforms to seven core symptoms: salience, mood modification, tolerance, withdrawal, conflict, problems, and relapse (Griffiths, 2005; Grant et al., 2010). This paper uses the Andreassen’s (2015) definition of SNS addiction: “being overly concerned about SNSs, to be driven by a strong motivation to log on to or use SNSs, and to devote so much time and effort to SNSs that impair other social activities, studies/jobs, interpersonal relationships, and/or psychological health and well-being.”

In the study of addiction disorders, psychological craving refers to the impulse to conduct addictive behavior that one cannot restrain, which is a subjective motivational state or a subjective desire or experience that individuals want to repeat. Psychological craving is one of the core features of addictive disorders because it is an observable clinical symptom which can explain an individual’s impulsive addiction behavior (American Psychiatric Association DSM- Task Force, 2013). It is one of the core issues that researchers have studied. According to the time and inducing factors, there were two types of craving: background craving and episodic craving (Shiffman, 2000), and the episodic was one of the main reasons for the failure of withdrawal. A study by Allen et al. (2008) showed that withdrawal was significantly positively correlated with craving. Meta-analysis studied the relationship between individual withdrawal and craving. The results suggested that a reduction in withdrawal symptoms led to a decrease in craving (Carter and Tiffany, 1999; Taylor et al., 2007). Psychological craving is the mechanism that forms and sustains an addiction disorder, causing the addict to be unable to stop due to withdrawal symptoms (Chase et al., 2011; Tiffany and Wray, 2012; Welberg, 2013).

Within the theory of craving, stability and continuity are controversial concepts. In terms of stability, previous studies hypothesized that the craving was stable for a certain period of time, while others hypothesized that the craving was constantly fluctuating (Marlatt, 1990; Davies et al., 2000). In terms of continuity, previous studies hypothesized that there were only two states of craving: existence and nonexistence (Kozlowski and Wilkinson, 1987). However, the empirical results showed that most people had craving in a continuous state, but different people had different levels of craving (Franken, 2003). There is no solid theory yet on how cravings change and the literature on this topic is sparse.

In previous studies of psychological craving, the researchers typically utilized a cue-reactivity paradigm to induce craving with words or pictures (Carter and Tiffany, 2001; Kühn and Gallinat, 2011). For example, a study of gambling addiction showed that cue-reactivity can cause a gambling addict to react with a significant increase in craving (Crockford et al., 2005). According to the biopsychosocial framework for the etiology of addictions and the syndrome model of addiction (Shaffer et al., 2004; Griffiths, 2005), individuals with SNS addiction experience symptoms similar to those experienced by those who suffer from chemical and behavioral addictions. Psychological craving also plays an important role in the study of Internet addiction (Kuss and Lopez-Fernandez, 2016). A study by Niu et al. (2016) including 40 Internet users suggested that network literature stimulation can induce the craving of Internet users, and Internet addicts’ cravings may have been more intense. A study by Chen et al. (2009) including 32 students who often played violent games also showed that the violent-related cue game can lead to a significant increase in craving.

Furthermore, previous studies have focused their attention on the brain activity of addicts. An event-related potential (ERP) study provided physiological evidence that excessive gamers showed significant differences in event-related potentials evoked by computer game-related cues at parietal regions (Thalemann et al., 2007). Previous research also examined crave-related cerebral regions with functional magnetic resonance imaging (fMRI). In one such study on game addiction that consisted of 10 game addicts and 10 healthy subjects, the researchers found that in the crave-related brain areas (dorsolateral prefrontal cortex, anterior cingulate cortex, right inferior parietal lobe), increased imaging signal densities were significantly and positively correlated with the craving scale scores (Sun et al., 2012). Another fMRI study of SNS addiction showed that gray matter volume is positively correlated with one’s level of SNS addiction, suggesting that the physiological basis of social network addiction may be found in gray matter (He et al., 2017).

Additionally, Brand et al. (2016) put forward a new theoretical model about specific Internet-use disorders named Interaction of Person-Affect-Cognition-Execution (I-PACE) model. In his theoretical model, individuals performing special network activities depend on a person’s core characteristics, subjectively perceived situation, affective and cognitive responses. An individual with Internet addiction receives gratification by using specific network functions (SNS addicts use WeChat) and the addict gratification is reinforced by the use of the Internet, resulting in the addict’s predispositions to receive gratification. According to the theoretical model, the gratification of individuals using SNSs strengthens the excitability of using SNSs, which leads to SNS addiction. Excitability can also be studied by the cue-reactivity paradigm. In the case of learning mechanisms, the reward effect of drugs became associated with addiction cues (Robinson and Berridge, 2008; Berridge et al., 2009). The cue-reactivity is accompanied by subjective scores and physiological responses, which can reflect the rewarding characteristics of potential factors to IAD (Carter and Tiffany, 1999; Stippekohl et al., 2010).

A review of the previous research of IAD indicates that there is little research on social network addiction and production of related desire (Kuss and Griffiths, 2011). Changes in the craving, excitability, or behaviors of Internet addicts are rare. Therefore, the effect of different materials on desire and excitability of social network addicts was measured in this study. The current study was divided into two experiments. The material of Experiment 1 is text, and the material of Experiment 2 is picture. The cue-reactivity is adopted to study whether the stimulation related to social network can improve the level of craving and excitability of individuals with SNS addiction. Additionally, the change in subjects’ craving level will be recorded.

## Materials and Methods

### Participants

We use Internet Relationship Dependence Inventory (IRDI) and Different Types of Internet Addiction Scale for Undergraduates to assess individual social network addiction trends. IRDI was compiled by Qian et al. (2006) and consists of 27 questions with a reliability Cronbach *α* of 0.92. According to the measurement results, if the average score of the subject is less than 3, the subject is defined as a normal user, and if it is not less than 3.15, the subject is defined as a pathological user. Different Types of Internet Addiction Scale for Undergraduates was compiled by Zhou and Yang (2006) and consists of 20 questions with a reliability Cronbach *α* of 0.80–0.92.

Participants were recruited from LiaoNing Normal University through IRDI [*N* = 169; 81 males, 88 females; age = 19–31 years, mean age = 24.2 years; of the 200 participants who logged in to the inventory, 31 (15.5%) abandoned the inventory before completing it, which left data from 169 participants in our next analyses]. All participants were right-handed,without neurological and psychiatric disorders, and had normal and corrected-to-normal vision. They had never participated in similar experiments previously and provided informed consent. The experimental protocol was approved by the local ethics committee. All participants were provided with experimental informed consent and participated in the experiment after signing the informed consent form.

The 169 participants who completed the IRDI were required to complete Different Types of Internet Addiction Scale for Undergraduates. Sixty participants were recruited through the analysis of the scores of IRDI and Different Types of Internet Addiction Scale for Undergraduates (30 males, age = 21–28 years, mean age = 23.9 years) and were paid some money to complete this experiment. Thirty participants achieved scores higher than the standard deviation of the two questionnaires from the high-score group. Another 30 participants got lower scores than the standard deviation of the two questionnaires from the low-score group. The high-score group indicates the tendency of the SNS addiction tendency as more serious, and the low-score group represents the tendency of the SNS addiction tendency of the participants as ambiguous.

### Materials and Stimuli

We first used the open questionnaire and interview method to collect SNS-related words and neutral words to select appropriate experimental materials. Nineteen students were then asked to use Level-7-score to assess those words in order to measure the score of SNS-related, pleasure, familiarity, and arousal. We chose 28 high SNS-related words (like Wechat, Tencent, *M* = 5.77 ± 0.18) and 28 neutral words (like weekend, gift, *M* = 2.89 ± 0.72) as the material of Experiment 1. The *t* test result showed that the two groups of words had a significant difference in SNS-related score (*t* = 18.72, *p* < 0.001). And these words were balanced on the other dimensions. All of these words were made into a 1,280 × 720 pixel picture. Using the same procedure, we chose 28 high SNS-related images (*M* = 5.08 ± 0.34) and 28 neutral images (*M* = 2.22 ± 0.40) as the material of Experiment 2. The *t* test results showed that the two groups of words had a significant difference in the SNS-related score (*t* = −28.917, *p* < 0.001), and these words were equaled in amount by the words for pleasure, familiarity, and arousal. All of these pictures make a 300 × 514 pixel picture.

### Procedure

Cue-reactivity paradigm was used. All the cues were evaluated by each subject. Thirty subjects (15 high-score group subjects) were asked to complete Experiment 1 first, then Experiment 2 after 7 days. Another 30 were asked to complete Experiment 2 first and then Experiment 1 after 7 days.

During the preparation, participants sat in a comfortable chair with a distance of 70 cm from the computer central screen in the laboratory. Participants were then asked to (1) turn off the mobile phone or mute the phone tone; (2) to assess their relaxation out of a Level-7-score (if the score of their relaxation was less than six, the participants would be asked to do a 5-min muscle relaxation exercise and then reappraise relaxation); and (3) to assess the psychological craving of the SNS if they passed the relaxation test were asked to assess the psychological craving of the SNS.

**Figure 1:**
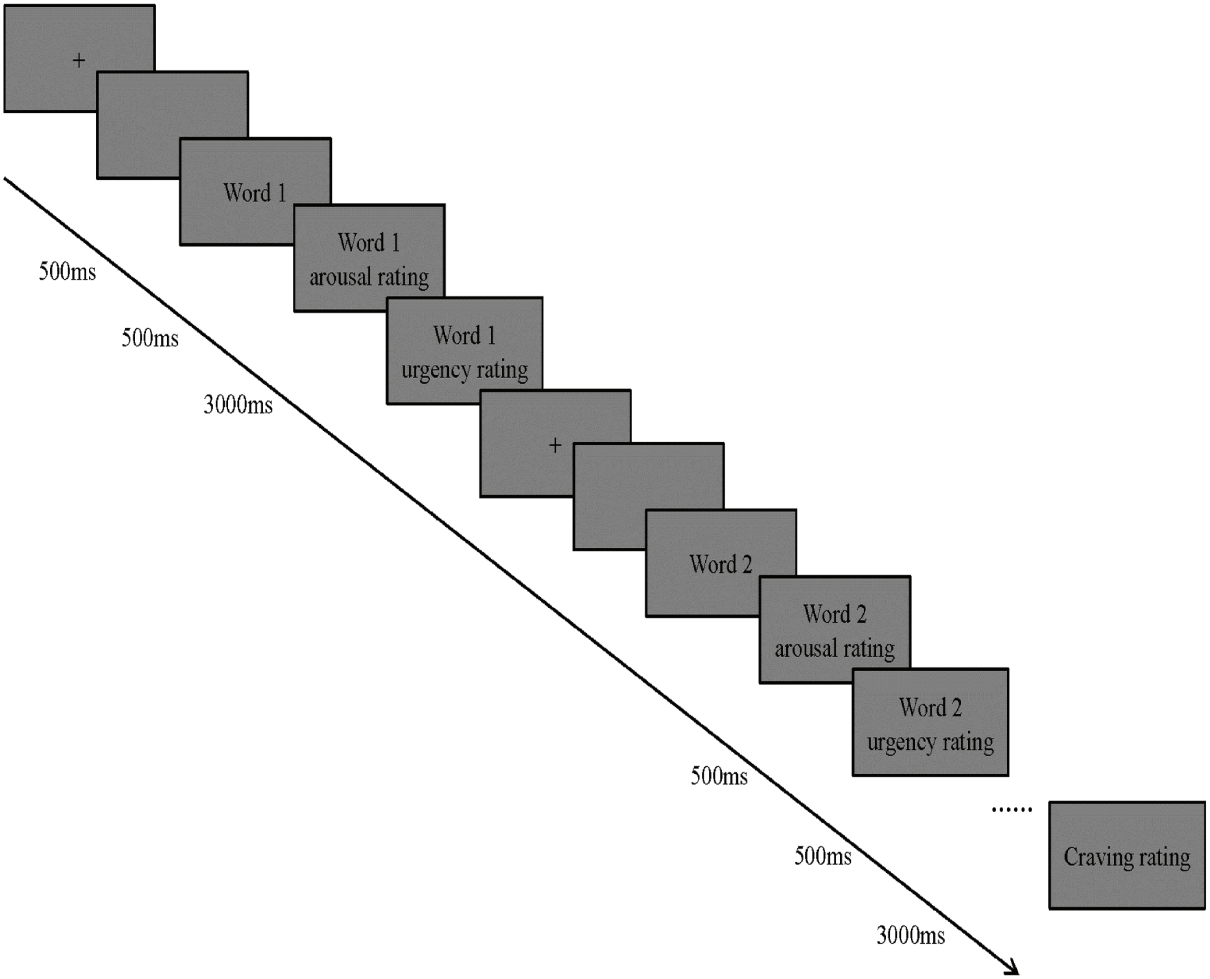
The paradigm used in the experiment.

In the experimental stage, the procedure based on E-prime vision 2.0 included two blocks, each block had 28 trials. At the beginning of the experiment, we showed the instruction in the middle of the screen, then asked participants to look at the pictures, carry on the free association, and then to assess each image for arousal and urgent degree score (Figure 1). The instructions explained to the participants the assessment rating of the arousal and urgent degrees.

The middle of the gray computer screen presented the fixation point 500 ms after the subject understood all the instructions and had pressed the space-bar. After that, the computer screen presented the empty screen for 500 ms, and then presented a random set of neutral clues (each group has seven pictures, four groups), each picture for 3,000 ms. The subjects were asked to perform free association tasks when stimulus was shown. To avoid potential induced effect, the experiment was organized so that neutral clues first appeared, before SNS-related clues were shown (Sayette et al., 2010). At the end of the neutral clue block, subjects had a mandatory 3-min rest time, and then were presented with a random set of SNS-related clue (each of the four groups had seven pictures). Presentation and evaluation of the neutral clues ran the same as with SNS-related clues. All SNS-related clue presentation and evaluation occurred after the last assessment after the test, at the end of the experiment.

## Result

### Social Network Craving

In Experiment 1, in order to judge whether the subjects were disturbed by external factors or not, we analyzed the effect of the muscle relaxation exercise. The result showed that the relaxation level of the high-score and low-score groups in the social network was relatively high. Moreover, there was no significant difference of relaxation level between the two groups (pre-test relaxation level: *t*(58) = 1.581, *p* = 0.119; post-test relaxation level: *t*(58) = −1.238, *p* = 0.221).

**Figure 2:**
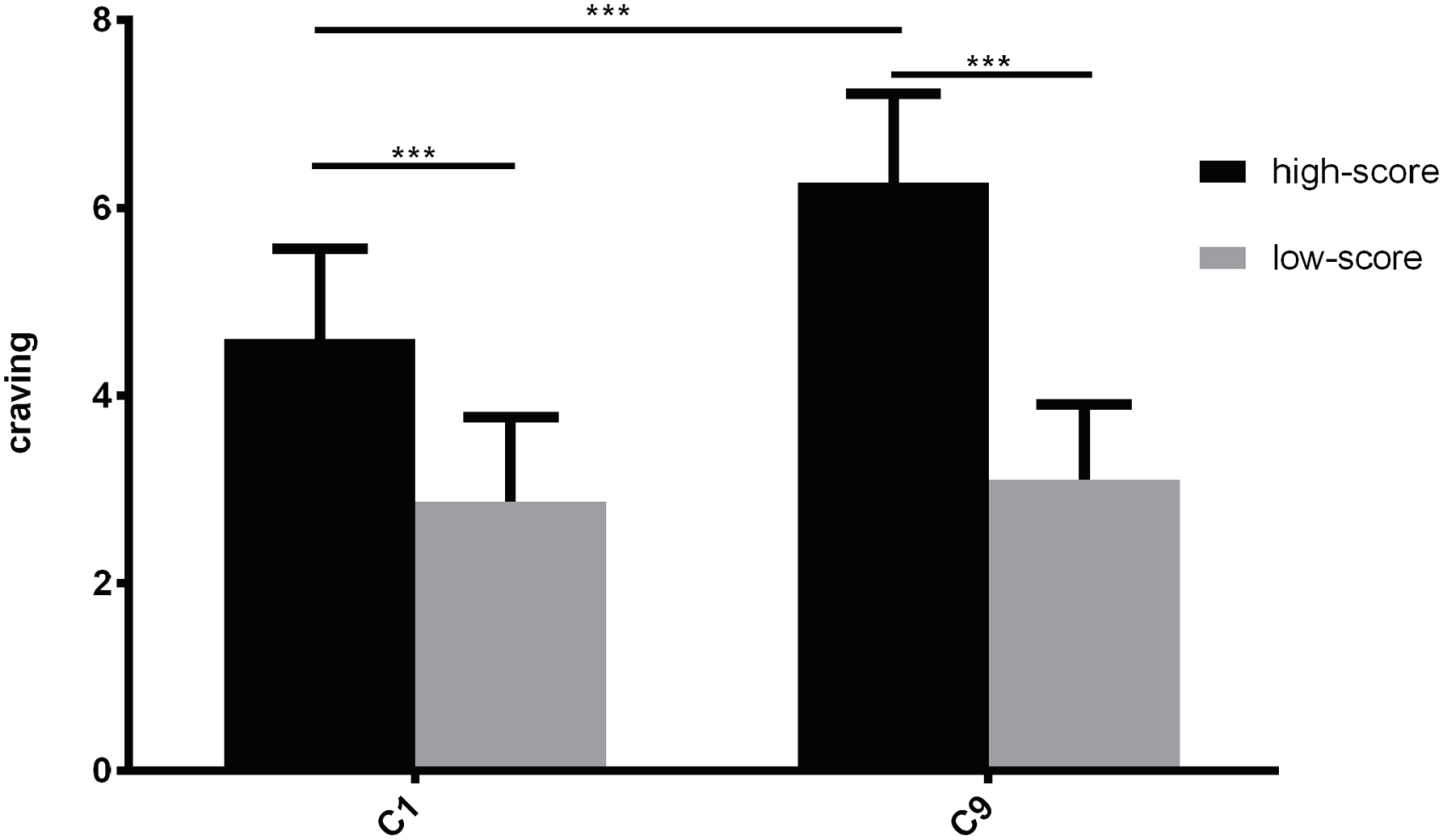
Comparison of pre- and post-craving measurements in Experiment 1. C1 is the pre-craving and C9 is post-craving. ***p < 0.001.

We analyzed the difference of craving post-test between the high-score and low-score groups. The results showed that the high-score group scored significantly higher than the low-score group in craving (*t*(58) = 13.991, *p* < 0.001). Moreover, in the high-score group, the post-test craving score was significantly higher than pre-test (*t* = −6.906, *p* < 0.001), while in the low-score group, it was not significant (*t* = −1.316, *p* > 0.1) (Figure 2).

**Figure 3:**
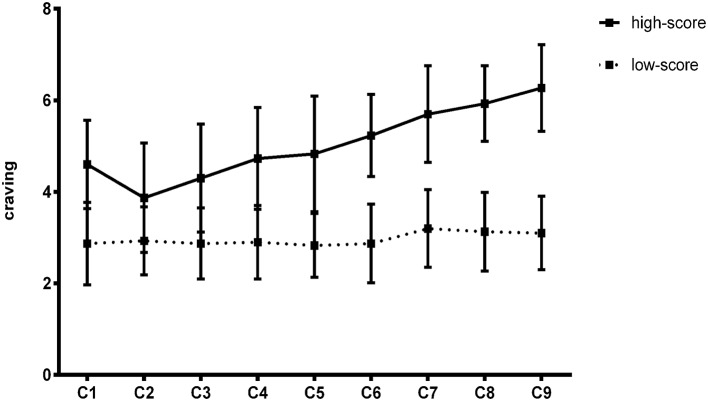
The change trend of craving in Experiment 1.

Comparing the changes in the psychological cravings of the two groups, the results showed that there was no significant difference in the psychological craving between the two groups when presented with neutral stimuli, but in the presentation of clue stimuli, the high-score group showed a gradual upward trend after the second test, and the low-score group approached no change (Figure 3).

Based on the above, we conducted a two-way AVONA (high-score group/low-score group × social network related/neutral). The results showed significant main effect of the group (*F*(1, 58) = 150.253, *p* < 0.001, $\eta_{p}^{2}$ = 0.721) and the clue type (*F*(1, 58) = 45.521, *p* < 0.001, $\eta_{p}^{2}$ = 0.440) as well as the group × clue type interaction (*F*(1, 58) = 21.439, *p* < 0.001, $\eta_{p}^{2}$ = 0.270). Due to the significant interaction effect, simple effect analysis was carried out to analyze the craving in different clue types. The results showed that the simple effect of the clue type in the high-score group was significant (*F*(1, 29) = 47.833, *p* < 0.001, $\eta_{p}^{2}$ = 0.623), while the simple effect of neutral stimulus in the low-score group is not significant (*F*(1, 29) = 3.463, *p* = 0.073, $\eta_{p}^{2}$ = 0.107). Results showed that for a high-score subject, craving induced by social network-related stimulus is significantly higher than neutral stimulus. But for the low-score group, there is no significant difference between the two types of clues.

In Experiment 2, in order to judge whether the subject was disturbed by external factors or not, we analyzed the effect of the muscle relaxation exercise. The results showed that the relaxation level of the high-score and low-score groups in social network was relatively high. Moreover, there was no significant difference of relaxation level between the two groups (pre-test relaxation level: *t*(58) = −1.758, *p* = 0.084; post-test relaxation level: *t*(58) = 0.266, *p* = 0.791).

**Figure 4:**
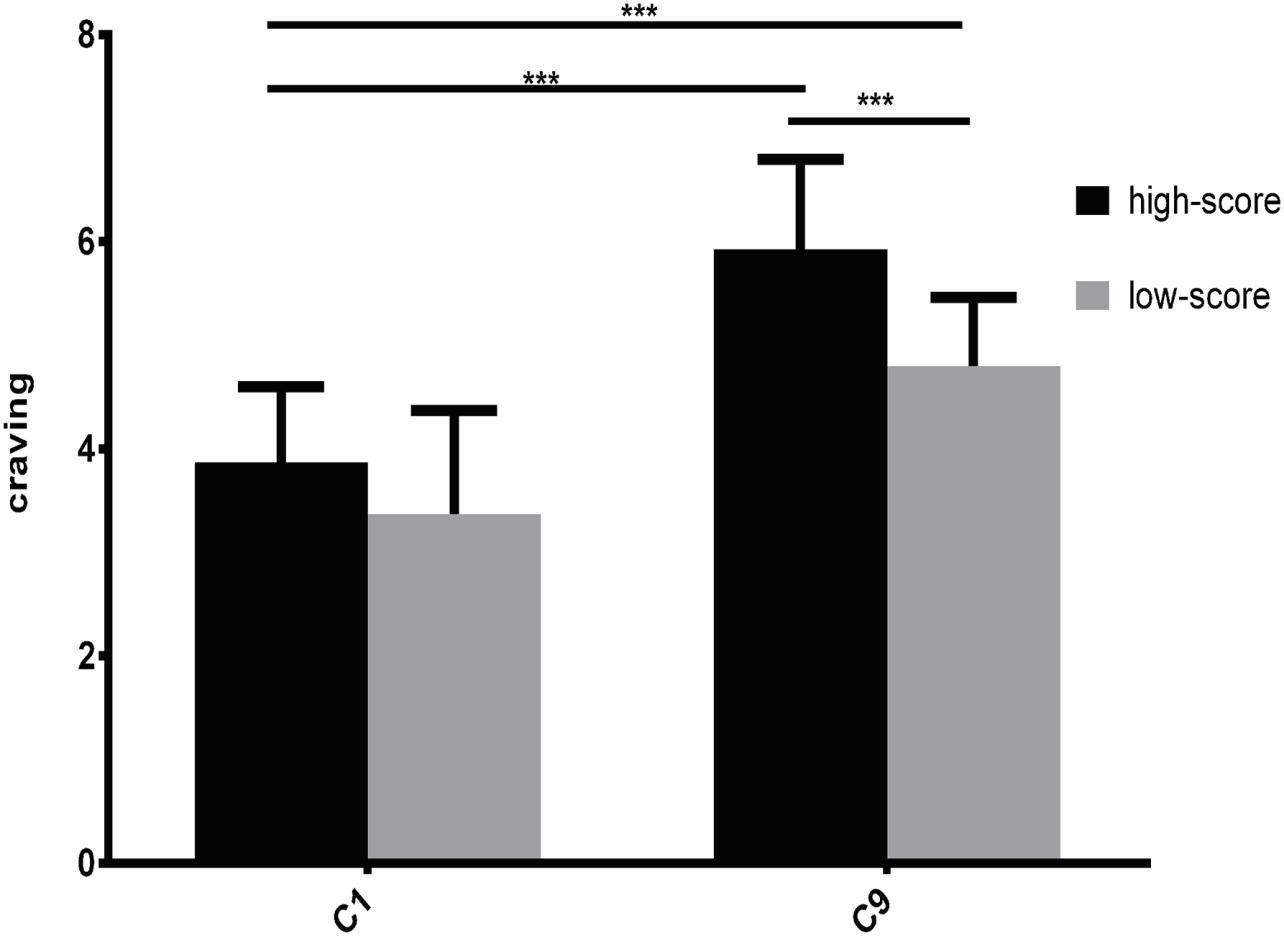
Comparison of pre- and post-craving measurements in Experiment 2. C1 is the pre-craving and C9 is post-craving. ***p < 0.001.

Then, we analyzed the difference of post-test craving between the high-score and low-score groups. The results showed that the high-score group was significantly higher than the low-score group in craving (*t*(58) = 5.678, *p* < 0.001). Moreover, the post-test craving was significantly higher than pre-test craving in all subjects (Figure 4).

**Figure 5:**
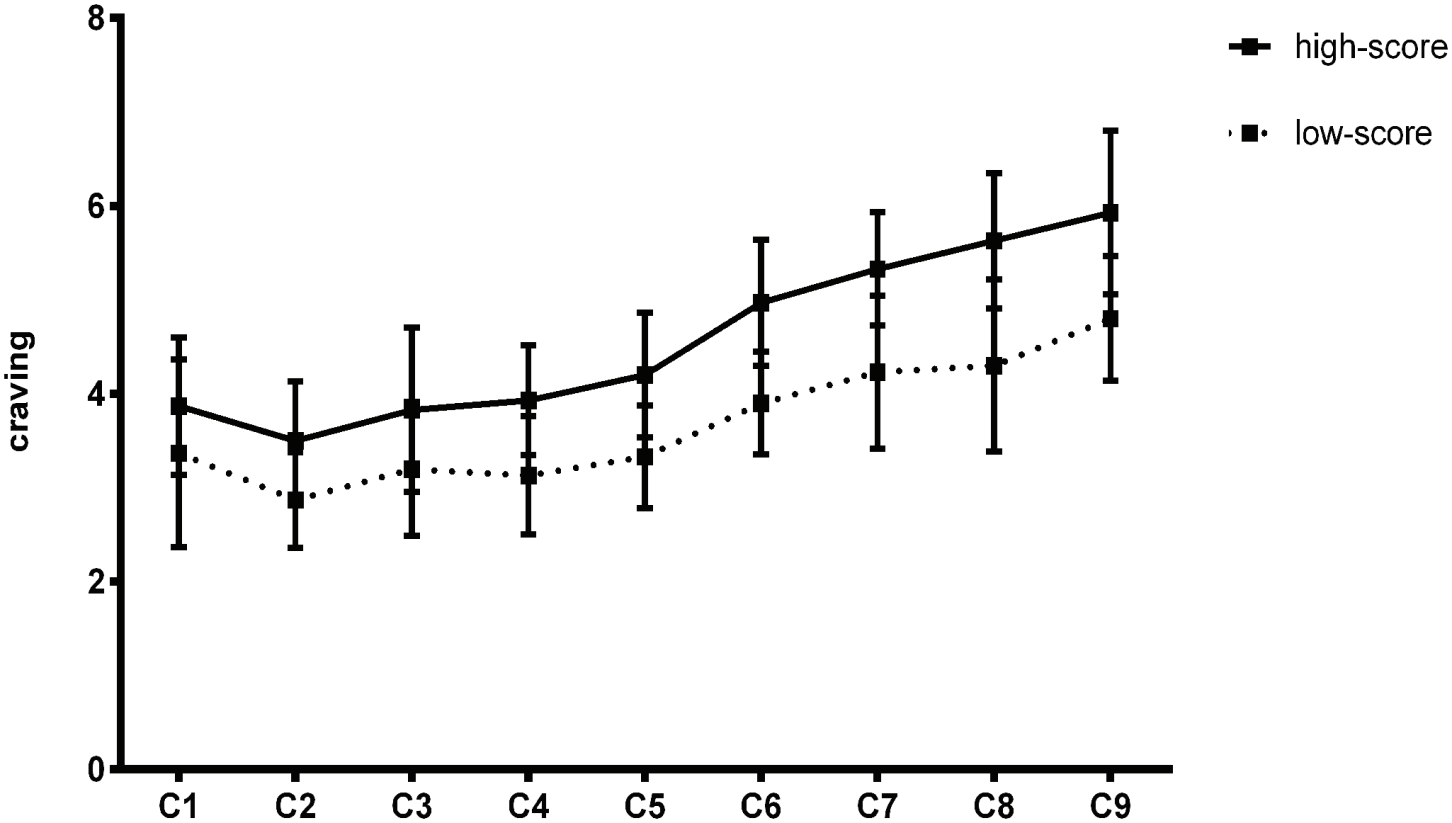
The change trend of craving in Experiment 2.

Comparing the changes in experimental material, the results showed that there was significant difference between the two groups when the material was an image, while there was significant difference in the high-score group but no significant difference in the low-score group when the material was text (Figure 5).

### Interaction Between Text and Picture

Based on the above, we conducted a two-way AVONA (high-score group/low-score group × image/text). The results of analysis of variance showed that the craving induced by social network use in the high-score group by social network-related images was significantly higher than with social network-related text (*F*(1, 58) = 94.477, *p* < 0.001, $\eta_{p}^{2}$ = 0.620). The low-score group also had a higher craving from social network-related images rather than text (*F*(1, 58) = 67.643, *p* < 0.001, $\eta_{p}^{2}$ = 0.538).

We conducted a three-way AVONA (high-score group/low-score group × social network related/neutral × image/text) (Table 1). The results showed that the simple effect of the clue type in the high-score group is significant (*F*(1, 29) = 114.540, *p* < 0.001, $\eta_{p}^{2}$ = 0.798), and the simple effect of the stimulating material in the high-score group was significant (*F*(1, 29) = 5.315, *p* < 0.05, $\eta_{p}^{2}$ = 0.155). The simple effect of the clue type in the low-score group was significant (*F*(1, 29) = 59.587, *p* < 0.001, $\eta_{p}^{2}$ = 0.673), and the simple effect of the stimulating material in the low-score group was significant (*F*(1, 29) = 47.419, *p* < 0.001, $\eta_{p}^{2}$ = 0.621).

**Table 1:** Three-way AVONA of craving.

	SS	df	*F*	$\eta_{p}^{2}$
Group	192.604	1	246.556***	0.810
Clue type	5.704	1	5.474*	0.086
Stimulus material	90.038	1	174.024***	0.750
Group × clue type	37.604	1	36.085***	0.384
Group × stimulus material	7.704	1	14.891***	0.204
Clue type × stimulus material	8.438	1	19.362***	0.250
Group × clue type × stimulus material	3.038	1	6.970*	0.107

*p < 0.05, ***p < 0.001.

We conclude that in the high-score group, social network-related clues can significantly induce the psychological cravings of using social networks, whether in the case of image or text, and in the case of images, the induced psychological craving is stronger. However, in low-score groups when using the image, compared with text, they have significantly more craving. Therefore, image stimulation can induce a stronger psychological craving than text stimulation.

### Social Network Excitability

In Experiment 1 of the present study, the social network excitability obtained from subjective scoring is the excitability of the participants in the social network-related text stimulation. After participants scored the arousal and urgency of some clue stimulus, we conducted a *t* test between scores of clue stimulus and neutral stimulus for the high-score and low-score groups. The results showed that the arousal and urgency score of either high-score or low-score groups from clue stimulus was significantly higher than neutral stimulus (Table 2).

**Table 2:** Analysis of excitability.

	High-score group	Low-score group	*t*
Arousal of SNS-related	3.69	2.76	−8.986***
Urgency of SNS-related	3.60	2.50	−9.201***
Arousal of neutral	2.90	2.95	0.379
Urgency of neutral	2.79	2.71	−0.713

***p < 0.001.

Based on the above analysis, we conducted a two-way AVONA (high-score group/low-score group × social network related/neutral) on the arousal and urgency of the subjects, respectively. The results showed that for arousal, the main effect of the group was significant (*F*(1, 58) = 18.929, *p* < 0.001, $\eta_{p}^{2}$ = 0.246), and the main effect of the clue type was significant (*F*(1, 58) = 23.269, *p* < 0.001, $\eta_{p}^{2}$ = 0.286), as well as the group × clue type interaction (*F*(1, 58) = 61.501, *p* < 0.001, $\eta_{p}^{2}$ = 0.515). For urgency, the main effect of the subjects was significant (*F*(1, 58) = 39.689, *p* < 0.001, $\eta_{p}^{2}$ = 0.406), and the main effect of the clue type was significant (*F*(1, 58) = 24.494, *p* < 0.001, $\eta_{p}^{2}$ = 0.297), as well as group × clue type interaction (*F*(1, 58) = 72.635, *p* < 0.001, $\eta_{p}^{2}$ = 0.556).

Due to the significant interaction effect, simple effect analysis was carried out to analyze the effect of different clue types on the arousal and urgency scoring of high-score and low-score groups. The results showed that the simple effect of the group in the arousal scoring was significant (*F*(1, 58) = 80.745, *p* < 0.001, $\eta_{p}^{2}$ = 0.582), and in the neutral text was not significant (*F*(1, 58) = 0.144, *p* = 0.706, $\eta_{p}^{2}$ = 0.002). The simple effect of the urgency scoring was significant (*F*(1, 58) = 84.650, *p* < 0.001, $\eta_{p}^{2}$ = 0.593), and in neutral text was not significant (*F*(1, 58) = 0.508, *p* = 0.479, $\eta_{p}^{2}$ = 0.009).

We conclude that social network-related texts can significantly induce the social network excitability of subjects. The high-score group was more excitable than the low-score group. The excitement induced by social network-related texts in high-score group is significantly high than that of low-score group.

In Experiment 2, the social network excitability obtained from subjective scoring is the excitability of the participants in the social network-related text stimulation. After participants scored the arousal and urgency of some clue stimulus, we conducted a *t* test between scores of clue stimulus and neutral stimulus for the high-score group and the low-score group. The results showed that the arousal and urgency scores of either the high-score group or the low-score group in clue stimulus were significantly higher than neutral stimulus (Table 3).

**Table 3:** Analysis of excitability.

	High-score group	Low-score group	*t*
Arousal of SNS-related	3.51	3.13	−3.698***
Urgency of SNS-related	3.56	3.19	−3.853***
Arousal of neutral	2.82	2.76	−0.848
Urgency of neutral	2.77	2.74	−0.383

***p < 0.001.

We conducted two-way AVONA (high-score group/social network related/neutral) for the arousal and urgency of the subjects. The results show that for arousal degree, the main effect of the subject group is significant (*F*(1, 58) = 9.139, *p* < 0.01, $\eta_{p}^{2}$ = 0.136), the main effect of the clue type is significant (*F*(1, 58) = 109.712, *p* < 0.001, $\eta_{p}^{2}$ = 0.654), and group × clue type interaction was significant as well (*F*(1, 58) = 9.474, *p* < 0.01, $\eta_{p}^{2}$ = 140). For urgency, the main effects of the subject group were significant (*F*(1, 58) = 10.808, *p* < 0.01, $\eta_{p}^{2}$ = 0.157), the main effect of the clue type was significant (*F*(1, 58) = 115.818, *p* < 0.001, $\eta_{p}^{2}$ = 0.666), and group × clue type interaction was significant as well (*F*(1, 58) = 8.971, *p* < 0.01, $\eta_{p}^{2}$ = 0.134).

The results showed that, compared with the neutral image stimulus, social network-related image stimulus can significantly induce the social network excitability of the subject; the social network of the high-score participants is more excitable than the low-score subjects. The excitability intensity of stimulus-induced high-score group social networks was significantly higher than that of low-score group.

We conducted three-way AVONA (Group × Clue type × Stimulus material) for the arousal and urgency of the subjects (Tables 4, 5). The results showed that the simple effect of clue type on the high-score group was significant (*F*(1, 29) = 172.762, *p* < 0.001, $\eta_{p}^{2}$ = 0.856), the simple effect of the stimulus material on the high-score group was not significant (*F*(1, 29) = 2.343, *p* = 0.137, $\eta_{p}^{2}$ = 0.075); the simple effect of the clue type on the low-score group was not significant (*F*(1, 29) = 2.944, *p* = 0.097, $\eta_{p}^{2}$ = 0.092), and the simple effect of the stimulus material on the low-score group was not significant (*F*(1, 29) = 1.087, *p* = 0.306, $\eta_{p}^{2}$ = 0.036). This result indicated that the high-score group had a significantly higher arousal when assessing social network-related stimulus than the arousal of neutral stimulus. However, there was no significant difference in the arousal scores of the high-score group subjects for different stimulus. For low-score subjects, the difference in arousal between the stimulus material and the type of clues was not significant.

**Table 4:** Three-way AVONA on arousal.

	SS	df	*F*	$\eta_{p}^{2}$
Group	6.478	1	26.835***	0.316
Clue type	10.417	1	112.596***	0.660
Stimulus material	0.026	1	0.118	0.002
Group × clue type	6.245	1	67.504***	0.538
Group × stimulus material	0.728	1	3.310	0.054
Clue type × stimulus material	0.808	1	7.957**	0.121
Group × clue type × stimulus material	1.655	1	16.289***	0.219

**p < 0.01, ***p < 0.001.

**Table 5:** Three-way AVONA on urgency.

	SS	df	*F*	$\eta_{p}^{2}$
Group	2132.509	1	59.556***	0.507
Clue type	12.691	1	118.251***	0.671
Stimulus material	1.600	1	7.429**	0.114
Group × clue type	7.111	1	66.256***	0.553
Group × stimulus material	2.278	1	10.577**	0.154
Clue type × stimulus material	1.542	1	15.084***	0.206
Group × clue type × stimulus material	1.768	1	17.287***	0.230

**p < 0.01, ***p < 0.001.

Further analysis of the effects of different clue types and stimuli on the urgency scores of the subjects was performed. The simple effect of clue types on high-score groups was significant (*F*(1, 29) = 132.370, *p* < 0.001, $\eta_{p}^{2}$ = 0.820), the simple effect of the stimulus material on high-score group was not significant (*F*(1, 29) = 0.124, *p* = 0.728, $\eta_{p}^{2}$ = 0.004); the simple effect of the clue type on the low-score group was not significant (*F*(1, 29) = 5.894, *p* < 0.05, $\eta_{p}^{2}$ = 0.169), and the simple effect of the stimulus material on the low-score group was significant (*F*(1, 29) = 20.328, *p* < 0.001, $\eta_{p}^{2}$ = 0.412). This indicates that the type of clue affects the urgency score of the high-score group but does not affect the low-score group, while the stimulus material type affects the urgency score of the low-score group but does not affect the high-score group.

### Discussion

#### Social Network Craving

The results of Experiment 1 showed that the social network-related stimulation could significantly induce subjects for the desire of social networks, and the score of high-score group is significantly higher than the low-score group. The results of our study are consistent with other addiction disorder research in that the external addiction-related stimuli could significantly evoke psychological craving (Tavares et al., 2005; Sinha and Li, 2007; Goudriaan et al., 2010). On the other hand, this study shows that SNS addiction and other addictive disorders (gambling, drug dependence, etc.) have a similar generation mechanism.

The incentive-sensitization model explains the mechanism of psychological craving. The sensitivity of the nervous system may be influenced by long-term addictive behavior and, therefore, becomes gradually more sensitive to addiction clues. The pathological features are called neural sensitization (Robinson and Berridge, 1993, 2001, 2008). Essentially, addicts will distort or exaggerate the incentive effect and direct attention toward addiction clues. Addiction clues arise as a result of the expected, causing addiction-related behavior or experiences. Social networks, as common communication tools in daily life, could induce the desire to use social networks in normal users, but for the addict could induce a desire for even more use of social networks.

In addition, the psychological craving as one of the core characteristics of addictive disorders is not isolated, but rather a subjective continuous motivation mode (Carter and Tiffany, 1999; Tiffany and Wray, 2012). Experimental results fully support this point in that for both social networking addicts and normal users, SNS clues will induce the psychological craving and lead the high-score groups to score even higher on measures of psychological craving.

In previous studies, researchers only measured the pre-test psychological craving, evoked psychological craving, and the post-test psychological craving. This study shows the change trend of the psychological craving of the participants. The high-score groups have an unapparent trend of rising and falling when the neutral stimulus is present. It also confirmed that the psychological craving is a kind of subjective motive of continuous model.

The results of Experiment 2 showed that the score of high-score group was significantly higher than the low-score group in craving (*t*(58) = 5.678, *p* < 0.001). The results of Experiment 2 again support the results of Experiment 1, which showed that SNS-related clues can successfully induce the craving of social addicts.

However, different types of clues lead to different effects of inducing the craving. In the high-score group, the image-induced craving was significantly stronger than the word-induced craving. In the low-score group, the cravings were also significantly increased when the images were used. Different stimulus materials affect individual cognitive processing. For example, the text stimulation affects the associative process while the image stimulation affects the associative and perceptual process (Li and Zhang, 2007). Therefore, under the stimulation of the image, the individual cognitive processing speed is faster and the level is deeper. When the individuals with SNS addiction saw the SNS-related picture, they noticed more details and were more likely to recall the pleasure of social network using, thus inducing a stronger craving. Similarly, because SNSs are a daily tool, the craving of the low-score group increased significantly when the images were stimulated.

#### Social Network Excitability

The study results showed that the social network-related stimulation could significantly induce an excitability of social networks in subjects and that the high-score group scored significantly higher than low-score group. The individuals with SNS addiction have a specific predisposition (high excitability) which makes it more likely that an individual uses social network sites because he/she anticipates excitability and gratification (Robinson and Berridge, 2000, 2003; Davis, 2001). Therefore, the high-score group experienced a greater level of social network excitability.

The results of Experiment 2 showed that there was no difference in the high-score group’s excitability, but the low-score group’s excitability was different in urgency. According to results, excitability is not a craving. This supports the theoretical model of I-PACE which posits that excitability is a predisposition that can increase the probability that an individual receives gratification from the use of certain applications and overuse (Brand et al., 2016). According to this model, excitability is enhanced during using. In our experiment, the subjects did not actually use social networks, so the change in excitability was not significant.

## General Discussion

In general, social networking is used as a daily social tool and individuals have a happy and satisfying experience for different needs and motivations. The formation of SNS addiction is a process of strengthening formation and maintenance because individuals are overly addicted to the experience and they overuse social networking sites. In this study, the results showed that the SNS-related stimulation could significantly induce the craving of social networks in subjects. This result is also in line with the positive reinforcement theory of craving – addicts establish a strong link between addiction-related stimuli and positive experiences of addictive behavior, and addiction-related stimuli can become prominent inducers and activate addictive memories which make it possible to anticipate the positive consequences of addictive behavior, and thus have the effect of inducing the craving of addicts (Sayette et al., 2000). The study found that social network-related stimuli can be used as effective stimuli to induce social network addicts’ cravings, which also validates the incentive-sensitization model in social network addict groups. Social network addicts have neural sensitization to social network addiction-related stimuli. The study of chemical and behavioral addictions showed that the cues associated with addiction significantly increased the cravings of addicts (Tavares et al., 2005; Sinha and Li, 2007; Goudriaan et al., 2010).

Additionally, previous research found that there are two kinds of nervous systems in the brain that exist in heroin addicts when they do cue-reactivity paradigm (Liu et al., 2011). The two kinds of nervous system are reward-circuits and mirror-neuron systems (Prisciandaro et al., 2012; Volkow et al., 2012; Yalachkov et al., 2012; Hanuschkin et al., 2013; Liu et al., 2013; Zeng et al., 2013). Similarly, research found that subjects, who have been smoking for a long time, exhibit the neural correlates of spontaneously activated action representations when subjects watch movie characters smoke (Wagner et al., 2011; Yalachkov and Naumer, 2011), indicating that addicts are watching addiction-related clues will activate the reward-circuits and mirror-neuron system to produce psychological craving. Moreover, Cheng et al. (2007) found that food-related stimuli elicited specific hemodynamic response in the mirror-neuron system, when subjects were in a hungry state as compared with a satiated state. Taken together, the production of psychological craving is related to the brain’s reward-circuits and mirror-neuron system. Therefore, the reward-circuits and mirror-neuron system of the brain are activated and produce cravings when individuals with SNS addiction see cues, but the cues do not activate the brains of non-addicts.

The results of Experiment 2 showed that the image-induced craving was significantly stronger than the word-induced craving. Different stimulus materials affect individual different cognitive processing, the text stimulation affects the associative process, and the image stimulation affects the associative and perceptual process (Li and Zhang, 2007). Under the stimulation of the image, the individual cognitive processing speed is faster and the level is deeper. The results are consistent with studies of mobile phone dependency that indicate that images are more stimulating than text stimulation is more effective. Moreover, there is a dual-process model showing that the image information is specific and intuitive, and the result of the image information processing is more emotional and more likely to trigger the memory of the subject (Sloman, 1996; Smith and DeCoster, 2000; Schnotz and Bannert, 2003; Osman, 2004). Therefore, the addicts were more likely to awaken the addiction-related memory when they looked at the image clues, and the memory meant the action observing neuron (AON) had a stronger response (Wagner et al., 2011).

Psychological craving changing tendency of the high-score group increases linearly. The craving of the low-score group is stable in Experiment 1 and also increases linearly in Experiment 2. The main causes of craving change in this study are the types of clues and intensity of clues. All the subjects were tested on computers, and computers were often used as operating platforms for social networking sites. The operation of computer is also an SNS-related clue that affects the craving changes of the subjects, so the craving change curve shows a linear increment.

The results also support that cravings were continuous but varied in intensity and therefore psychological craving fluctuates (Franken, 2003). The craving induced by addiction-related clues is a kind of neural activity in the activation mode (Marlatt, 1990; Davies et al., 2000). Both the priming effect and the level of neural activity in the brain affected the level of craving. The level of neural activity is also affected by many factors.

SNS-related stimuli can induce an individual’s excitability but the type of cue cannot affect the individual’s excitability. According to the theoretical model of I-PACE (Brand et al., 2016), the SNS addict’s excitability increases the probability that an individual receives gratification from the use of SNS and overuses these applications again (Brand et al., 2011; Kuss and Griffiths, 2011; Pawlikowski et al., 2013, 2014). The excitability is the emotional and dynamic foundation of the craving in this model, and the cues associated with addiction make individuals more aroused. But excitability is enhanced during using. In our experiment, the subjects did not actually use social networks, so the change in excitability was not significant.

## Conclusions

In the current study, we documented cravings and excitability induced by different clues and obtained evidence that the SNS-related clues can induce the craving and excitability of the individuals with SNS addiction. Our findings support the hypothesis that different materials have different effects of cravings, and the effect of image clues was significantly higher than that of text clues. The cravings of the individuals with SNS addiction are constantly fluctuating. These results have theoretical implications for learning more about the mechanisms of social networking sites addiction.

## Data Availability

All datasets generated for this study are included in the manuscript and/or the supplementary files.

## Ethics Statement

All the subjects were paid volunteers and gave their written informed consent. This study was approved by Liaoning Normal University Human Research Institutional Review Board in accordance with the Declaration of Helsinki (1964). All of the subjects were healthy adults, and we confirm that no minors, persons with disabilities or endangered animal species participated in the study.

## Author Contributions

WH designed the experiment. YL, XH, BZ, and WH wrote the paper. PL and CX collected the data. Besides, all authors reviewed the manuscript.

### Conflict of Interest Statement

The authors declare that the research was conducted in the absence of any commercial or financial relationships that could be construed as a potential conflict of interest.
